# Young women’s perceptions of cervical screening in the UK: a qualitative study

**DOI:** 10.1017/S1463423624000446

**Published:** 2024-10-18

**Authors:** Monique Taratula-Lyons, Marie C. Hill

**Affiliations:** 1 City St. George’s, University of London., London, UK; 2 School of Health & Psychological Sciences, City St. George’s, University of London., London, UK

**Keywords:** Cervical cancer screening, barriers, facilitators, qualitative research, interviews, women’s health

## Abstract

**Aim:**

To understand young women’s views of cervical screening, what obstacles they face, and what encourages them when considering attending their cervical screening.

**Background:**

Cervical screening figures have been steadily decreasing in the United Kingdom (UK). There is limited research on this trend, especially around views and knowledge of young women, aged 20–24 years, have before they are eligible for cervical screening.

**Methods:**

This qualitative study conducted 15 semi-structured Zoom in-depth interviews to discuss young women’s knowledge and perceptions of cervical screening in 2022. Participants were based in the UK. Thematic analysis was used to systematically manage, analyse, and identify themes including cervical screening knowledge; perceptions of cervical screening; barriers to cervical screening; and facilitators of cervical screening.

**Findings:**

The findings demonstrate significant gaps in knowledge and negative perceptions of cervical screening. Barriers to attending cervical screening were perceived pain and embarrassment. Facilitators suggested to promote attendance were ensuring access to appointments, creating pop-up clinics, and utilising incentives. The level of knowledge demonstrated by the participants, their negatively framed perceptions; and the vast number of barriers identified present substantial factors that could affect future attendance to cervical screening. Overall, action needs to be taken to prevent decreasing cervical screening attendance rates and eradicate any barriers women may experience.

## Background

This section explores what cervical screening is, the global rates of cervical cancer, screenings in the United Kingdom (UK), and young women’s views of cervical screening and introduces the aim and the research questions of this study.

### What is cervical screening?

Screening is defined as assessing a healthy population to identify those at risk or with signs of disease (WHO, 2022). Screening aims to reduce the chance of a disease developing within a section of the population; therefore, screening can save lives (Aschengrau and Seage, 2018). However, it is dependent on an individual’s choice to attend a screening appointment (Aschengrau and Seage, 2018).

Cervical screening aims to detect human papillomavirus (HPV), which is a common and easily contracted sexually transmitted infection (Roland *et al*., 2013). There are several types of HPV, which live on the skin and are often transferred by sexual penetration, although HPV can still develop without having sexual intercourse (NHS, 2022). HPV can lead to cervical, vaginal, and vulva cancer (NHS, 2022). However, many HPV infections do not present any complications, with 90% of cases being cleared within two years, and infection can be prevented by using condoms (NHS, 2022).

Roland *et al*. (2013) describe cervical screening as a routine test for women where endocervical cells are taken from the cervix. If no HPV cells are present in the cervix, no further action is necessary. However, HPV may be found with no abnormal cells being located, indicating that supplementary screenings may be required (Roland *et al*., 2013; NHS, 2022). If HPV and abnormal cells are detected, further tests, such as a colposcopy may be essential, including treatment before the cells develop into cancer (NHS, 2022).

### Global cervical cancer rates

Cervical cancer is prevalent around the world, in 2022, cervical cancer had an incidence rate of 662,301 million cases globally, making it the eighth highest compared to other types of cancer (Ferlay *et al*., 2024). However, women living in low- and middle-income countries have the highest cervical cancer incidence and mortality rates (WHO, 2024). This is due to underlying inequalities and lacking access to cervical cancer screening, treatment, and the HPV vaccination (WHO, 2024). Cervical screening has decreased the number of deaths arising from cervical cancer, demonstrating its importance in reducing global cervical cancer rates (Mayor, 2016).

### Cervical screening in the UK

In the UK, approximately 854 people die from cervical cancer every year (Cancer Research UK, 2022). Cervical screening was first introduced in the UK in 1964 and is currently offered to anyone with a cervix aged 25–64 years, every 3–5 years (NHS, 2022). Due to the selected sample, cisgender women will be the focus of this research.

From 2022 to 2023, of 4.62 million individuals invited for their cervical screening in the UK, only 3.43 million women aged 25–64 years were screened (NHS Digital, 2023). This is a decrease of 2% from the previous year when approximately 3.50 million individuals attended their cervical screening (NHS Digital, 2023). Figures for cervical screening in 2021 present that almost one-third (30%) of individuals eligible were not screened, with one in three women in the UK not attending their appointments (Department of Health and Social Care, 2022). Furthermore, if everyone eligible for a cervical screening attended regularly, it is estimated that 83% of cervical cancer cases could be prevented (Public Health England, 2019). This indicates that factors hinder the attendance of cervical screening.

### Young women’s views of cervical screening

Women aged 25–29 years are less likely to attend their screening appointments and attendance has been declining since 2014 (Waller *et al*., 2011; Public Health England, 2017). The most common reasons that prevent women from attending their screening appointments include thinking that it is not necessary and being embarrassed or fearful (Department of Health and Social Care, 2022). It is important to recognise that women eligible for cervical screening face different barriers and, therefore, factors that may encourage attendance also need to be identified.

Survey results found that 59% of women aged between 25 and 29 years dislike the idea of cervical screening but see it as an essential test, 27% find the process embarrassing, and 21% experience pain during their screening (Jo’s Cervical Cancer Trust, 2017). Embarrassment was the main reason for not booking an appointment from a survey undertaken by the Department of Health and Social Care (Department of Health and Social Care, 2022).

Of a sample of 3,002 women aged between 25 and 29 years over two-thirds do not think cervical screening will reduce their risk of cervical cancer (Jo’s Cervical Cancer Trust, 2017). A literature review conducted in the UK has highlighted that 40% of women were able to correctly identify the symptoms of cervical cancer, with only 1% stating HPV as a cause (Foran and Brennan, 2015). Common symptoms include pain in your lower back, bleeding between periods, and changes to vaginal discharge (NHS, 2021). Therefore, a lack of knowledge surrounding cervical cancer and cervical screening appears to inhibit attendance. This indicates the need for education curricula to cover the importance of cervical screening, what occurs during the appointment, as well as safe sex practices to reduce HPV, and ultimately prevent cervical cancer.

Health promotion theory can be applied to this topic to gain an alternative perspective. The Health Belief model is one of the most influential models, developed in 1966 by Irwin Rosenstock, it involves four constructs and is used to predict general and positive health behaviours (Raingruber, 2017). A main critique of this model is that it relies on individual factors (Roden, 2004). However, this can be a beneficial way to incorporate a framework in this topic, as it can focus on individual views and barriers. In Singapore, as part of a Healthy Living Master Plan, a health promotion framework has been applied to holistically view and alter health issues to overcome barriers (Ministry of Health, 2014). This includes the 3Ps, place, people, and price, aiming to collectively create an inclusive, affordable environment for healthy living (Ministry of Health, 2014). Therefore, incorporating a model within this topic would help focus on individual views and develop ways of reducing obstacles to cervical screening, while creating a rationale to support behaviour change.

The crux of the issue of low attendance at cervical screening interlinks with a lack of knowledge combined with fear and embarrassment (Jo’s Cervical Cancer Trust, 2017). This demonstrates how action needs to be taken to improve women’s knowledge and views of cervical screening before they are eligible for their appointments.

Women aged 25–29 years are the group least likely to attend cervical screening (Public Health England, 2017). Previous research about cervical screening has often lacked qualitative studies that attempt to comprehend what may prevent and encourage women to attend, especially before they are offered an appointment (Foran and Brennan, 2015; Mayor, 2016). This empirical research will seek to fill this gap by investigating the knowledge and views of young women, between the age of 20–24 years, through semi-structured interviews.

The aim and research questions that this study intends to answer are as follows:

Aim:

- To understand young women’s views of cervical screening, what obstacles they face, and what encourages them when considering to attending their cervical screening.

Research questions:

- What are young women’s views of cervical screening?
- What is preventing and or encouraging young women to attend their cervical screening?

## Methods

The following section covers the method utilised for the study, ethical considerations, and tools that are used to enhance rigour and reflexivity.

### Design

This research collected primary qualitative data to understand the views young women have about cervical screening. Denzin and Lincoln (2008) discuss how qualitative data is essential in determining the deeper meaning of a topic, allowing an individual’s experiences to be considered and discover the crux of an issue (Denzin and Lincoln, 2008). However, qualitative data can be time-consuming to undertake and is often nuanced (McCusker and Gunaydin, 2014).

### Study setting and recruitment

Before the recruitment process began, research into the necessary sample size to reach data saturation took place. Guidance from Francis *et al*. (2010) indicates that 10 semi-structured interviews, followed by another 3 if necessary, ensure that a topic is comprehensively investigated, provided no supplementary themes are identified in the additional interviews. Fifteen interviews were conducted online, to allow for cancellations and technical difficulties.

Participants were identified, approached, and recruited by using the social media platform Instagram as it attracts a young demographic which was desirable for this research (Catalani and Hughes, 2020). An Instagram account was created, and a post was published to allow potentially interested participants to contact the researcher.

This research used two non-probability samples that interlink: convenience and snowball sampling to ensure the desired sample size was reached. Convenience sampling allows anyone who volunteers and fits the criteria to participate in the study and is commonly used when recruiting participants online (Galloway, 2005). However, convenience sampling is vulnerable to bias, as the sample is not representative of the entire population (Galloway, 2005). The second method selected, snowball sampling, is beneficial for recruiting participants on social media by utilising social connections, as posts can be shared amongst individuals (Wright and Stein, 2005). The researcher used snowball sampling by sharing the post that was published on Instagram and encouraged their network to circulate the post to anyone who may be interested in participating in the study. Similar to convenience sampling, this method often relies on social connections where a level of bias is inherent (Galloway, 2005; Wright and Stein, 2005).

Semi-structured interviews were chosen to ensure a level of privacy around this topic and allow participants to freely discuss their knowledge and perception of cervical screening (Francis *et al*., 2010).

### Inclusion and exclusion criteria

The study recruited female participants, aged between 20 and 24 years. Only participants who had not yet received their invitation to attend their first cervical screening appointments were included. This is because the research aimed to understand young women’s perceptions of cervical screening before they are invited to attend their appointments. This excluded men, women not aged between 20 and 24 years and women who had already had a cervical screening. Additionally, this study excluded those who cannot speak English and who did not live in the UK.

### Data collection

The interviews were held online in February and March 2022, using Zoom to interview participants across the UK. Thirteen questions were asked during the interviews covering two categories: knowledge and views of cervical screening (see Appendix 1). Each interview lasted between 15 and 40 min, with interviews being recorded and transcribed verbatim.

### Data analysis

Thematic analysis was selected as the analytical tool for this research, as it is an efficient way of establishing parallels and patterns across transcripts (Braun and Clarke, 2006). Firstly, the interviews were checked for accuracy. Following this, M. T. L. read all the transcripts to solidify their understanding of what was covered in the interviews. Keywords and quotes were collected in a document to keep track of any emerging themes, and these were condensed to the most commonly occurring themes. During this process, subthemes were identified, quotes were highlighted in colours corresponding to a theme, and a spreadsheet was created to present the quotes for each theme.

### Ethical considerations

Ethical approval was gained by the University Maternal and Child Health Proportionate Review Committee (14/02/2022; Reference number: ETH2122-0683).

A participant information sheet was sent via email to potential participants once they acknowledged their interest with the researcher, to assist in their decision to take part in the study. Participants also received a consent form, which required a digital signature and a signed copy to be sent back to the researcher, before an interview date was arranged.

Maintaining confidentiality is a substantial ethical issue. Mechanisms were put in place to ensure that all data collected were protected: by conducting a risk assessment before the research commenced, immediately anonymising any personal data collected and storing the data on a password-protected computer only known to the researcher. In order to minimize potential risks to the research participants, the data were made anonymous by allocating a number to each participant. Moreover, due to elements of the subject matter being deemed sensitive, the researcher collated a list of charities to enable any participant who disclosed previous trauma to access support. Any personal data were kept in accordance with the Data Protection Act 2018 and the General Data Protection Regulations.

### Rigour and reflexivity

Personal reflexivity highlights the researcher as a vital and visible aspect of the research process (Olmos-Vega, Stalmeijer, Varpio and Kahlke, 2022). Furthermore, subjectivity acknowledges how both participants and researchers bring their histories, politics, and assumptions into research (Sharp, 2020). It is crucial to understand and identify aspects of the researcher’s characteristics that may have influenced this research. A checklist to ensure the trustworthiness and credibility of the research project can be seen in Appendix 2.

As M. T. L. is a woman, they may have been less objective, and their feminist views may have affected the data collected. However, this may have allowed participants to feel more open to sharing their views than if a male researcher had interviewed them.

Both convenience and snowball sampling rely on the researcher’s pre-existing social connections (Wright and Stein, 2005). This resulted in recruiting some participants who were known to M. T. L. and who potentially had similarly high levels of education, introducing a level of bias. Conversely, participants known to the researcher appeared to be more comfortable talking in-depth about cervical screening, the discussions of which likely revealed information that, otherwise, might not have been elicited.

## Findings

**Figure 1.**
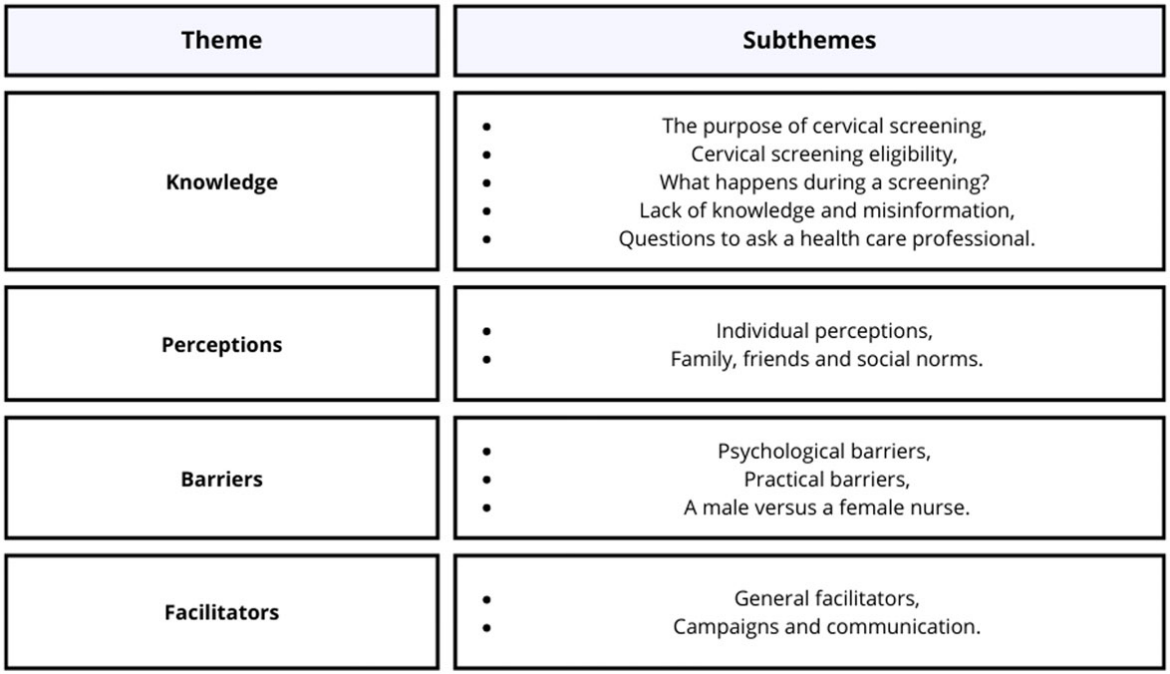
The themes are: Knowledge (subthemes: The purpose of cervical screening, cervical screen eligibility, what happens during a screening? lack of knowledge and misinformation and questions to ask a health care professional), Perceptions (subthemes: individual perceptions, family, friends and social norms), Barriers (subthemes: Psychological barriers, practical barriers and a male versus female nurse) and facilitators (subthemes: General facilitators, campaigns and communication).

Four key themes were identified following the semi-structured interviews, namely: cervical screening knowledge, perceptions of cervical screening, barriers to cervical screening, and facilitators to cervical screening including sub-themes within each theme. See Figure 1 for a visual representation of all themes and sub-themes.

### Characteristics of participants

The 15 semi-structured interviews were conducted with women, with the majority aged 23 years and residing in a range of locations within the UK.

### Cervical screening knowledge

Several sub-themes emerged from this theme, such as the purpose of cervical screening, cervical screening eligibility, what happens during a screening, lack of knowledge and misinformation, and, lastly, questions that participants wish to ask a healthcare professional.

#### Purpose of cervical screening

The interview discussions first centred around the purpose of having routine cervical screening. The quotes below are examples that demonstrate the varying knowledge participants had about cervical screening. The majority of participants stated that screening was to detect cancer, and HPV was occasionally brought up as a purpose of cervical screening. However, there appeared to be some confusion about the relationship between cervical cancer and HPV, with participants lacking knowledge about the purpose of cervical screening and what the tests entail.

‘They check them for either cancer or HPV. I don’t know which one.’

P1 (aged 22).

‘It’s always been something I’m aware of, but I’ve never really known exactly what they’re looking for or what they’re testing.’

P7 (aged 22).

‘So, it’s to detect any changes in the cells of your cervix, that might be sort of pre-warning towards cervical cancer, I think.’

P13 (aged 23).

#### Cervical screening eligibility

The interviews then explored women’s eligibility for cervical screening. Most participants were aware of the general age range for eligibility for a screening and had a vague knowledge of the frequency of attendance. There was uncertainty of the exact facts, and questions were raised about whether an individual had to be sexually active to be eligible for cervical screening. The frequency of appointments was often unknown, with some participants’ suggesting that screening happens once a lifetime or every year.

‘I think it’s just once. But I might be wrong.’

P2 (aged 20).

‘I think it’s supposed to be women over the age of 24, or maybe 25 I can’t quite remember.’

P8 (aged 23).

‘I thought it was only for like sexually active people.’

P9 (aged 24).

#### What happens during a screening?

Cervical screening was described by the participants in varying degrees of detail. There appeared to be a rudimental understanding of what occurs during an appointment. Although the main elements of cervical screening were often known by the participants, uncertainty can lead to misinformation, which could be detrimental to cervical screening attendance.

‘I think it’s like collect cells, or like get a little…it sounds gross but a scraping, or like collect cells or something that they can examine.’

P10 (aged 24).

‘They put something up your vagina. And then the nurse does some sort of swabby thing up your vagina.’

P3 (aged 23).

‘I had the vaccine when I was in school, so I know that that’s something to do with it.’

P7 (aged 22).

#### Lack of knowledge and misinformation

Throughout the interviews, there were areas of knowledge that could be improved. There was a lack of clarity around the different terms used in cervical screening, and what occurs during the appointment. For instance, attendance at cervical screening was perceived as mandatory.

‘I don’t know I would just kind of say that you have to.’

P8 (aged 23).

‘And then like what are they going to do to you? Cut out your uterus? How does it work?’

P3 (aged 23).

‘With a knowledge gap normally comes misinformation, because people fill that gap with something that works for their agenda. And so if we don’t have the full and the factual information, people will feel the gaps with what works for them.’

P7 (aged 22).

#### Questions to ask a healthcare professional

During the interviews, participants were asked what questions they had about cervical screening. Many participants questioned why the procedure was so painful. Participants stated that they wanted a truthful response from a healthcare professional, so they were aware of what happens during a screening and thus could mentally prepare before they went for an appointment.

‘What can we do as people to reduce the fear of cervical cancer screening?’

P11 (aged 23).

‘How it feels to have the screening done, is it super intrusive, is it uncomfortable?’

P15 (aged 23).

‘How uncomfortable or painful it is. If there are any kind of innovations going on with cervical screenings.’

P12 (aged 23).

### Perceptions of cervical screening

For this theme, the sub-themes include individual perceptions, those of family and friends and social norms.

#### Individual perceptions

Most participants conveyed a negative view, despite having an underlying understanding that cervical screening is essential for health. Individual experiences were mentioned which appeared to improve understanding of cervical screening and indicated that a lack of experience, personal or anecdotal, may heighten anxiety. For instance, one participant discussed getting their intrauterine device (IUD) fitted and how they felt less apprehensive about internal examinations after their appointments.

‘Before I had the IUD fitted, I would have actually been like absolutely not, I don’t want anyone to see my vagina, and I think it would have made me feel like really anxious and just like apprehensive.’

P4 (aged 23).

‘Imagine something like quite clinical and quite cold and quite unpleasant.’

P1 (aged 22).

#### Family and friends and social norms

Conversations with family and friends about cervical screening appeared important to the participants’ understanding of this process. Most participants acknowledged that screening was a personal choice, although many would encourage eligible family members and friends to attend. However, discussions participants had with family and friends were represented as brief, as cervical screening was seen as a stigmatised and personal topic. Many participants said they would not feel comfortable discussing cervical screening with family members and may be more inclined to talk to friends. Some participants raised how their age affected their dialogue when discussing cervical screening.

‘I don’t really feel like anyone talks about screening like this. Maybe that’s because I’m just not old enough yet.’

P8 (aged 23).

‘I think it’s a taboo, taboo-ish type thing to talk about…even within women it’s not discussed that much.’

P10 (aged 24).

### Barriers

The barriers identified by participants included: psychological barriers, practical barriers, and whether having a male or female nurse is a barrier to attendance.

#### Psychological barriers

The most common psychological barriers mentioned were anxiety, awkwardness, and embarrassment. Participants mentioned being nervous and apprehensive about their first screening. The impact of certain ‘horrific’ shared experiences was seen as likely to prevent future attendance.

‘I would say again it probably just comes back to my nervousness about it.’

P10 (aged 24).

‘Previous bad experiences would definitely put you off, I know if I didn’t know a bit more about the process, like if someone told me a horror story about it, it would definitely put me off.’

P13 (aged 23).

#### Practical barriers

Other significant barriers to cervical screening appointments were practical barriers, such as appointment times and location, as well as working hours. Participants mentioned how their job might affect their attendance.

‘Lots of doctor’s surgeries are only open from 9-5, which for someone like me, I wouldn’t be able to get there at those hours.’

P3 (aged 23).

‘It depends on how long it would take and certain factors that could prevent you, travelling far distances just to do the screening.’

P15 (aged 23).

#### A male nurse versus a female nurse

Many participants were not perturbed by the prospect of a male nurse if the individual felt comfortable in the environment. Conversely, some participants mentioned how a male nurse may make them feel more anxious and could pose a barrier to attendance. The reasoning for this was due to the belief that a female nurse would understand the procedure on a higher emotional and personal level compared to a male nurse.

‘I don’t know if a male could properly, properly understand.’

P10 (aged 24).

### Facilitators

Several facilitators were identified by participants to promote cervical screening, including general facilitators, campaigns, and communication.

#### General facilitators

The main facilitator for attending cervical screening was to improve individual health, which took priority for many over any pre-existing anxiety. Ideas such as pop-up clinics were mentioned as a method to overcome inflexible appointments and inaccessible General Practitioner surgeries. Incentives utilising food and money were another facilitator discussed to increase screening attendance.

‘Like a pop-up clinic just for that, in somewhere like I don’t know it’s just somewhere local like that lots of people can get to. […] Maybe reimbursed for travel or things like that.’

P13 (aged 23).

‘Getting a chocolate bar at the end of it, that would definitely entice me to go, probably not most women but having some sort of like yay, you’ve done it.’

P3 (aged 23).

‘Outweighed by the importance of health.’

P4 (aged 23).

#### Campaigns and communication

The role of campaigns and communication was discussed as a crucial tool and potential facilitator to improve awareness and promote conversations and attendance at cervical screening. Many participants had only seen information circulated online, such as on Instagram or TikTok and through word of mouth, demonstrating little awareness of any NHS or national campaigns. Participants identified how certain strategies such as providing real-life experiences of cervical screening would be an asset to promote this service.

‘I can’t ever recall seeing an actual like you know NHS like ad or something being like remember to go for your cervical screening check.’

P9 (aged 24).

‘I have I’ve seen people like mainly on like Instagram, or like TikTok, like just videos like the process of going for a cervical screening.’

P13 (aged 23).

‘Personally, I think real people with real stories would probably be the most effective.’

P10 (aged 24).

## Discussion

This qualitative study explored young women’s perceptions and the level of knowledge that they have about cervical screening and identified the facilitators and barriers facing access to cervical screening.

### Knowledge about cervical screening

Often participants in the study mentioned HPV but did not link it as a cause of cervical cancer, demonstrating how their knowledge and understanding of cervical screening is minimal. The findings of this study align with research conducted by Foran and Brennan (2015) who highlighted that only 1% of their sample were aware that HPV is a cause of cervical cancer. Consequently, the abundance of questions asked by the participants demonstrates a significant knowledge gap, one that could be preventing attendance to life-saving cervical screening.

During the interviews, participants referred to the HPV vaccine, with some mentioning their experience getting the vaccine. In the UK, the HPV vaccine programme has been available in schools for girls aged 11–13 years since 2008 and has been offered to boys of the same age since 2019 (Public Health England, 2017). From 2008 to 2009, there was high uptake of the vaccine, with approximately 88% of females getting one dose and 80% receiving all three doses (Public Health England, 2015). The cohort of girls who were the first to be vaccinated are currently aged 25–29 years and are now eligible for their first and second cervical screening (Public Health England, 2017). The use of the vaccine has decreased cervical cancer incidence rates by approximately 87% for young women (Jo’s Cervical Cancer Trust, 2021). However, participants appeared to only have a vague recollection of receiving the vaccine and the information about cervical cancer and screenings they were given at the time.

In the UK, cervical screening is offered to those with a cervix, registered at a general practice, and aged between 25 and 64 years (NHS, 2022). Nevertheless, participants were not always certain about the age range that were covered by the cervical screening programme. Moreover, the number of questions raised by participants in the study-illustrated gaps in knowledge, with key questions centring around if an individual had to be sexually active to be eligible for cervical screening, which is not correct (NHS, 2022).

Cervical screening usually entails an examination of the cervix, where a sample is taken and sent for testing to detect any abnormalities (Roland *et al*., 2013). Nonetheless, only a few study participants were able to provide this level of detail. Furthermore, Waller *et al*. (2011) indicate that knowledge about cervical screening is limited for younger audiences and often develops the older the population becomes. Therefore, it is imperative for education curricula and NHS resources to cover cervical screening to increase attendance at appointments for younger generations.

### Perceptions

When discussing cervical screening perceptions during the study, many participants had pessimistic views about the screening process. However, there was an underlying understanding that cervical screening is essential for individual health, the necessity of which is acknowledged by Mayor (2016). The participants in the study often relied upon similar experiences, such as their IUD being fitted, or what family and friends had told them, to inform them about cervical screening. However, this does not appear to be a main factor in previous studies (Foran and Brennan, 2015; Momberg *et al*., 2017), demonstrating the need for further research.

### Barriers

Psychological barriers to attend cervical screening are prominent in previous research, affirming that most women are anxious, embarrassed, and concerned about the invasive nature of cervical screening (Jo’s Cervical Cancer Trust, 2017). This demonstrates that the apprehension and embarrassment associated with cervical screening are substantial psychological barriers. Furthermore, the emotions felt by individuals should be recognised and understood by the healthcare professionals undertaking the screening to ensure attendees are respected and looked after.

Practical barriers, such as working hours and appointment location and time, were another key aspect of this study. It was found that accessing screening and strict appointment times are prominent barriers for a younger audience (Waller *et al*., 2011). The debate about a male or female nurse conducting a cervical screening appointment was identified as a notable barrier in this study. Nevertheless, there is limited evidence that supports this finding. Conversely, some barriers can be removed easily by creating flexible appointments, inspiring discussions, and ensuring those eligible know that they can request a female nurse (NHS, 2022). In addition, by placing this study in the context of health promotion theory, barriers can be viewed alongside other factors, as well focusing on individual barriers (Raingruber, 2017).

### Facilitators

Facilitators identified in the study discussed suggestions to improve cervical screening rates, including incentives such as offering food or travel reimbursements. Financial incentives have been effective at influencing behaviour change, particularly to encourage attendance at appointments and screenings (Kane, Town and Butler, 2004; Sutherland, Christianson and Leatherman, 2008). In Singapore, the Healthy Living Master Plan was adopted and the 3Ps were developed in order to create a healthy environment (Ministry of Health, 2014). The third element of the 3Ps is price, where micro-financial incentives are utilised to nudge people to live a healthier lifestyle (Ministry of Health, 2014). However, using incentives may detract from the message that attending screenings is crucial for health. Therefore, communicating the essential nature of cervical screening for an individual’s health through a campaign could be a more successful, ethical, and cheaper solution (Tambor *et al*., 2016; Colleoni, Bartholomew and Schmidt, 2022).

Most participants gained information about cervical screening through word of mouth or via social media. The utilisation of campaigns on online platforms targeted at young women could increase cervical screening turnout. However, this will only be achieved by understanding the factors inhibiting young women from engaging with campaigns.

### Strengths and limitations of the work

The following section will discuss the limitations of this study and address additional elements that could be considered for further research.

Qualitative research provides a snapshot of the participant’s perceptions and, due to the small sample size, these findings are not generalisable (McCusker and Gunaydin, 2014). In addition, Zoom was selected as a separate video and audio recording was available. Occasionally, feedback or background noise distorted participants’ answers. Moreover, once the recording stopped, some participants’ nerves dissipated and they were able to share further thoughts on the topic that were not documented.

For future research, face-to-face interviews or the use of telephonic interviews should be considered to remove the risk of technical issues and could be a more effective method to gain full insight into the participant’s views. In addition, further research could compare perceptions across different age groups, participants of varying education levels, non-binary, and transgender participants or investigate male knowledge and perceptions of cervical screening.

## Conclusion

This study investigated young women’s views around cervical screening, determining what motivates them and deters them from considering attendance at cervical screening. It is found that the level of knowledge among participants was varied, with gaps identified that could be damaging to future attendance. While most participants felt compelled to attend their appointment despite lacking vital knowledge, this study has highlighted deficiencies in understanding why cervical screening is important, what they test for, and how this procedure takes place. Initiatives for younger generations should be a priority for schools and universities to ensure the necessity of cervical screening is communicated to younger women so that once they become eligible, they attend their appointments.

Various barriers to cervical screening attendance are prominent, particularly psychological factors regarding how invasive the procedure is, and the perceived pain involved. In addition, practical barriers were also significant, with many GP surgeries and appointments not perceived as being flexible or accessible (Public Health England, 2017). Further research into this topic is essential to combat barriers and negative perceptions.

Furthermore, facilitators such as incentives and communicating the importance of cervical screening from a health perspective should be highlighted as a method that could encourage young women to attend their appointments. Additionally, a health promotion framework should be applied to this topic to focus on individual motivations and barriers and adapt behaviour change within this topic. Overall, open discussions are necessary to comprehensively tackle the social norms and barriers young many women perceive to affect their attendance at the cervical screening.

## Supporting information

Taratula-Lyons and Hill supplementary material Taratula-Lyons and Hill supplementary material

## Supplementary material

To view supplementary material for this article, please visit https://doi.org/10.1017/S1463423624000446
