# Supplementary material for: Young women’s perceptions of cervical screening in the UK: a qualitative study
Source: Prim Health Care Res Dev. 2024 Oct 18;25:e49. doi: 10.1017/S1463423624000446 (PMC11569844; doi:10.1017/S1463423624000446)
Supplement: Taratula-Lyons and Hill supplementary material [file S1463423624000446sup001.docx]

### **Appendix 1: Interview Questions Guide**

**First section - Knowledge of cervical screening**

1. What do you know about cervical screening?
2. How would you describe cervical screening?
3. What do you think is the purpose of cervical screening?
4. Who should attend cervical screening and why?
5. How often do you think you have to attend a cervical screening? (Looking at the frequency of attendance).
6. Can you explain what you think may happen during a cervical screening?
7. Is there anything that you would like to know about cervical screening? (Any specific questions).

**Second section- Views of cervical screening**

1. When the words’ cervical screening is mentioned, how does this make you feel?
2. Would cervical screening be something you feel comfortable talking about with your family or friends?
3. Do you know anyone who has been to a cervical screening, did they share their experience?
4. Have you ever seen a campaign about cervical screening? If so, how did this make you feel?
5. What would encourage you to attend cervical screening?
6. What would prevent you from attending cervical screening?

### **Appendix 2: Checklist**

| **Item** | **Checklist** |
| --- | --- |
| **Title**- create a concise title  **Abstract**- provide a summary | ✔ |
| **Introduction:**  Problem formulation- description of the problem  Purpose or research question- purpose, objectives, questions | ✔ |
| **Methods:**  Qualitative approach and research paradigm  Researcher characteristics and reflexivity -personal characteristics Context  Sampling strategy/participant selection -sampling, sample size Ethical issues pertaining to human subjects  Data collection methods- the setting of data collection  Data collection instruments and technologies -interview guide, data saturation Units of study  Data processing  Data analysis - transcription, derivation of themes Techniques to enhance trustworthiness | ✔ |
| **Results/findings:**  Synthesis and interpretation - data and findings are consistent, and contain clarity of major and minor themes  Links to empirical data- quotations presented | ✔ |
| **Discussion:**  Integration with prior work, implications, transferability, and contribution to the field Limitations | ✔ |
| **Other:**  Conflicts of interest  Funding- sources of funding | ✔ |

(Adapted from: Tong et al., 2007; O’Brien et al., 2014).
